# Orogenic structure and topography track subduction singularities during slab delamination and detachment

**DOI:** 10.1038/s41598-025-94789-2

**Published:** 2025-04-09

**Authors:** Mark R. Handy

**Affiliations:** 1 Freie Universität Berlin, Berlin, Germany; 2 Universität Bern, Bern, Switzerland

**Keywords:** Subduction, Singularity, Orogenesis, Indentation, Drainage divide, Ocean sciences, Solid Earth sciences

## Abstract

A new model of Alpine mountain-building based on state-of-the-art seismic imaging explains how slab delamination and detachment facilitated indentation and led to along-strike changes in orogenic structure, denudation and basin dynamics. After Adria-Europe plate collision (40–32 Ma), slab steepening and delamination of the European slab changed the taper angle of the orogenic wedge in the Central Alps as the subduction singularity migrated northward. This induced rapid exhumation and denudation of the Lepontine orogenic core, accompanied by waves of clastic deposition in the overfilled western foreland basin. In the Eastern Alps, the heavier part of the slab delaminated further northward, driving prolonged subsidence and marine sedimentation in the underfilled eastern foreland basin. At ~ 20 Ma, the slab segment beneath the Eastern Alps detached, facilitating fragmentation of the indenting northern edge of the Adriatic Plate. This offset the collisional edifice while reorganizing subduction singularities and bifurcating drainage divides. Slab detachment triggered rapid uplift and terrigenous filling of the eastern foreland basin, together with orogen-parallel extrusion of the rapidly exhuming Tauern orogenic core toward the Pannonian Basin. There followed a dramatic shift in thrust-activity and -vergence from northward to southward. Similar lateral variations are documented for other orogens experiencing slab delamination and detachment.

## Introduction

**Fig. 1:**
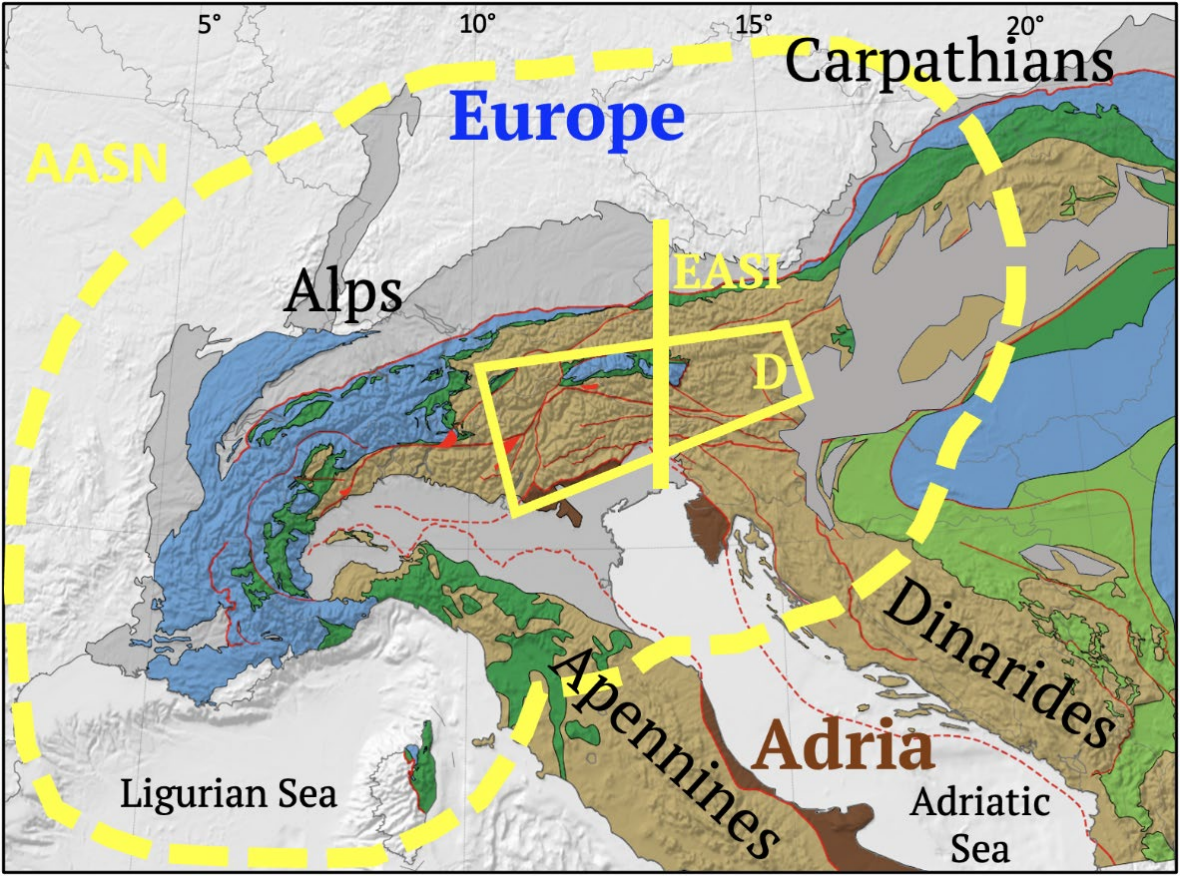
Tectonic map of the Alps with adjacent chains and basins. Colours indicate plate affinity: blue = accreted continental Europe, brown = accreted continental Adria, dark brown = autochthonous Adria. Other colours: dark grey = peripheral Tertiary basins (foreland Molasse Basin, hinterland Po-Veneto Basin, Pannonian Basin). Mesozoic sutures (dark green = Alpine Tethyan ophiolites, light green = Neotethyan ophiolites). Alpine Tethyan ophiolites mark the Adria-Europe plate boundary in Late Cretaceous-to-Eocene time (simplified from^2,3^). Yellow dashed line encloses AlpArray passive seismic network (AASN) comprising >600 broadband stations, yellow box outlines Swath D targeted array with 154 broadband stations, yellow line—EASI passive seismic swath.

The European Alps along the Adria-European plate collision zone (Fig. 1) are attractive to study mountain-building processes, partly because they have long been an archetype of orogenesis, but also because they are host to a wealth of modern studies that in past decades have targeted their subsurface. Recently, this has included *AlpArray*, a multidisciplinary passive-array campaign to illuminate crustal and mantle structure down to the mantle transition zone (^1^, Fig. 1).

**Fig. 2:**
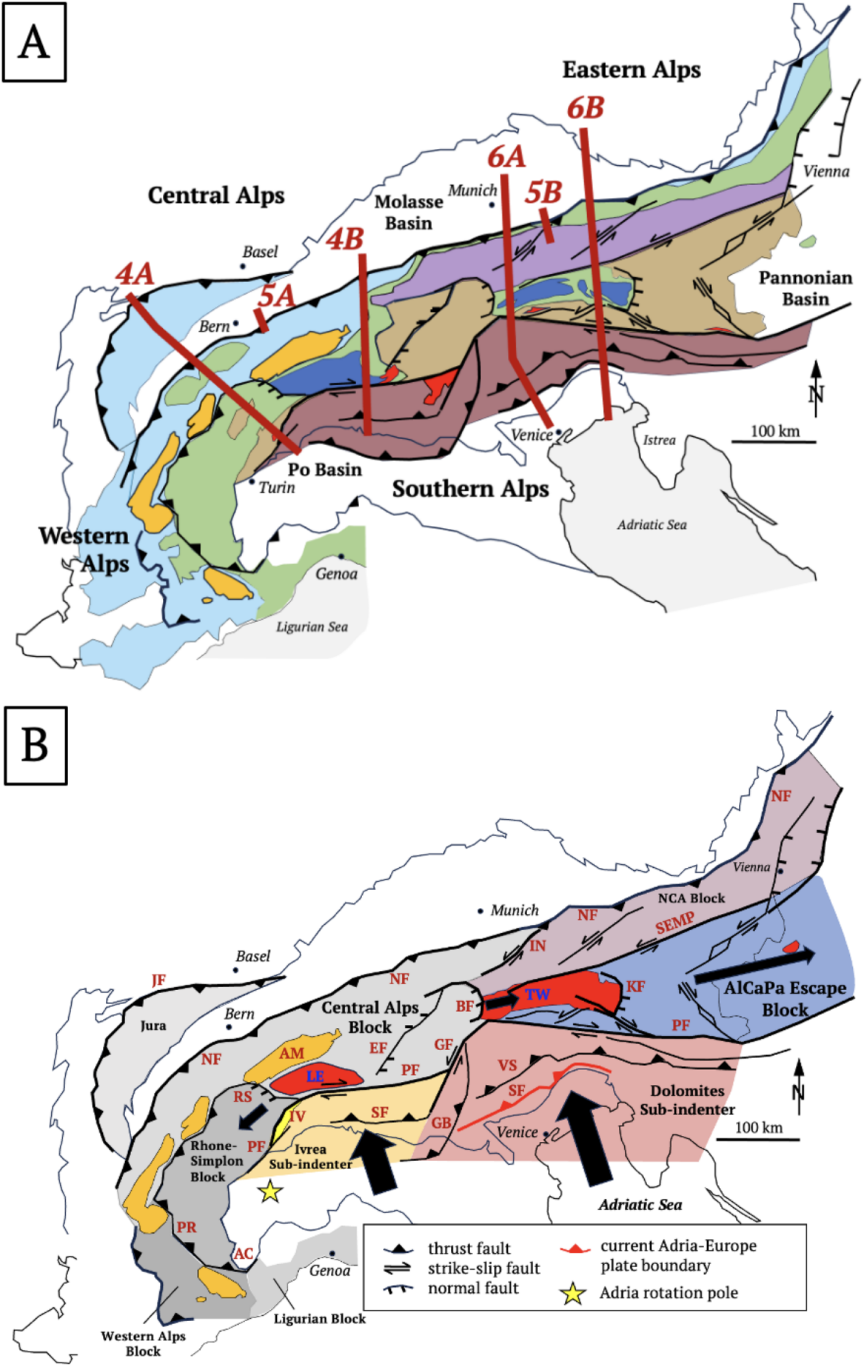
Tectonic overview: (**A**) Main tectonic units: blue—Helvetic Nappes & Jura fold-and-thrust belt; orange—External Basement Massifs; dark blue—Sub-Penninic units (European distal continental margin); green—Penninic units comprising Alpine Tethyan units (Valais, Piemont-Liguria ophiolites) and Briançonnais (European continental margin); light brown—Austro-Alpine Nappes; dark brown—Southern Alps (Adriatic continental margin); purple—Northern Calcareous Alps (NCA); red—Tertiary Periadriatic intrusives, enclosed white area—Cenozoic basins (Molasse, Po-Veneto, Pannonian). Brown lines—cross sections numbered according to Figures in text: 4A – NFP20W; 4B—NFP20E; 5A – Entlebuch; 5B – Perwang; 6A – TRANSALP; 6B – section parallel to EASI based on seismology and interpretations, respectively, in^14–19^; (**B**) Crustal blocks during Neogene-to-recent indentation: Indenting blocks at the deformed leading edge of the Adriatic Plate: Dolomites Subindenter (red-brown), Ivrea Subindenter (yellow–brown) including the Ivrea Zone, IZ (yellow). Thick arrows indicate Neogene motion of Adria with respect to Europe. Indentation increases eastward from the counterclockwise rotation pole (yellow star) of Adria with respect to Europe. Thin black arrows indicate motion of indented orogenic blocks with respect to Europe: AlCaPa Escape Block (blue) and Northern Calcareous Alps Block, NCA (violet). Indented orogenic crust of the Central Alps comprises the Central Alps Block, kinematically connected to the Jura fold- & thrust sheet. The Jura thrust front (JF) and Northern orogenic front (NF) bound the pro-wedge of the Alpine orogen. The Southern orogenic front (SF) bounds the retro-wedge. The Western Alps and Ligurian Blocks experienced counterclockwise oroclinal bending in Early-Mid-Tertiary time. Other indentational structures: orange – External Basement Massifs including the Aar Massif (AM); bright red—Tertiary post-nappe metamorphic domes (LE—Lepontine, TW—Tauern Window). Faults: AC—Argentera-Cuneo Fault, BF—Brenner Normal Fault, EF – Engadine Fault, GB – Giudicarie Belt, GF – Giudicarie Fault, JF – Jura thrust front, KF – Katschberg Normal Fault, NF – Northern orogenic front, PF—Periadriatic Fault, PR—Penninic Front, RS—Rhône-Simplon Fault System, SEMP—Salzach–Ennstal–Mariazell–Puchberg Fault, SF – Southern orogenic front (active segment marked red), VS – Valsugana Thrust. Thin black lines—borders of peripheral basins.

These studies reveal dramatic across- and along-strike variations in orogenic style, from “hard” collision in the Alps featuring Oligo-Miocene indentation by the Adriatic Plate, to “soft” collision in the Carpathians involving Miocene rollback subduction of the European Plate and opening of the Pannonian Basin^4,5^. Subsequently, Adriatic indentation fragmented the Alpine orogenic crust (Fig. 2A) into fault-bounded domains and structures that overprint subduction and collisional structures formed during Cretaceous-to-Paleogene time (Fig. 2B). Indentation resulted from slow 0.5 mm/a convergence and ~ 5° counterclockwise rotation of the Adriatic Plate with respect to Europe since at least 20 Ma^6^ about a pole in the western part of the Southern Alps (Fig. 2B, e, g.,^7,8^). Oroclinal bending of the Western Alps occurred earlier, mostly during late Cretaceous-to-late Paleogene Adria-Europe convergence^9^ and was accentuated by Neogene rollback subduction of the Adriatic Plate beneath the Apennines^10–12^. The Alps-Apennines Junction thus coincides with a switch in subduction polarity^13^, from European subduction beneath the Alps to Adriatic subduction beneath the Apennines (Fig. 1).

Debate centers on whether the along-strike variability of the Alps reflects pre-orogenic structure^2^, rheological heterogeneities in the mantle^20^, removal of subducted lithosphere^21,22^, a switch in subduction polarity^23^, indentation tectonics^24^ or some combination thereof. To distinguish these processes, we combine the results of *AlpArray* and a related multidisciplinary project (*Mountain-Building in 4-Dimensions, 4D-MB*) to track subduction singularities and major river drainage divides in the Alps through time.

A subduction singularity is an abrupt stress- and strain-rate discontinuity within mountain belts, below which lithosphere subducts and above which detached crust advects upwards, forming a topographic ridge or drainage divide at the surface^25^. In two-dimensional orogenic models, a subduction singularity and topographic ridge separate an elongate thrust-and-fold belt (pro-wedge) and foreland basin above the downgoing plate from a narrower, steeper belt with opposite thrust vergence (retro-wedge) and pronounced exhumation adjacent to a hinterland basin on the upper plate^26^. The location of subduction singularities and drainage divides is sensitive to the rates and amounts of subduction, accretion and denudation, as well as to lithospheric heterogeneity. Here, we show how shifting subduction singularities and drainage divides in the Alps are related to partial slab detachment and indentation, effecting orogen-parallel changes in thrust activity, vergence and basin dynamics.

## Results

### Mantle structure

**Fig. 3:**
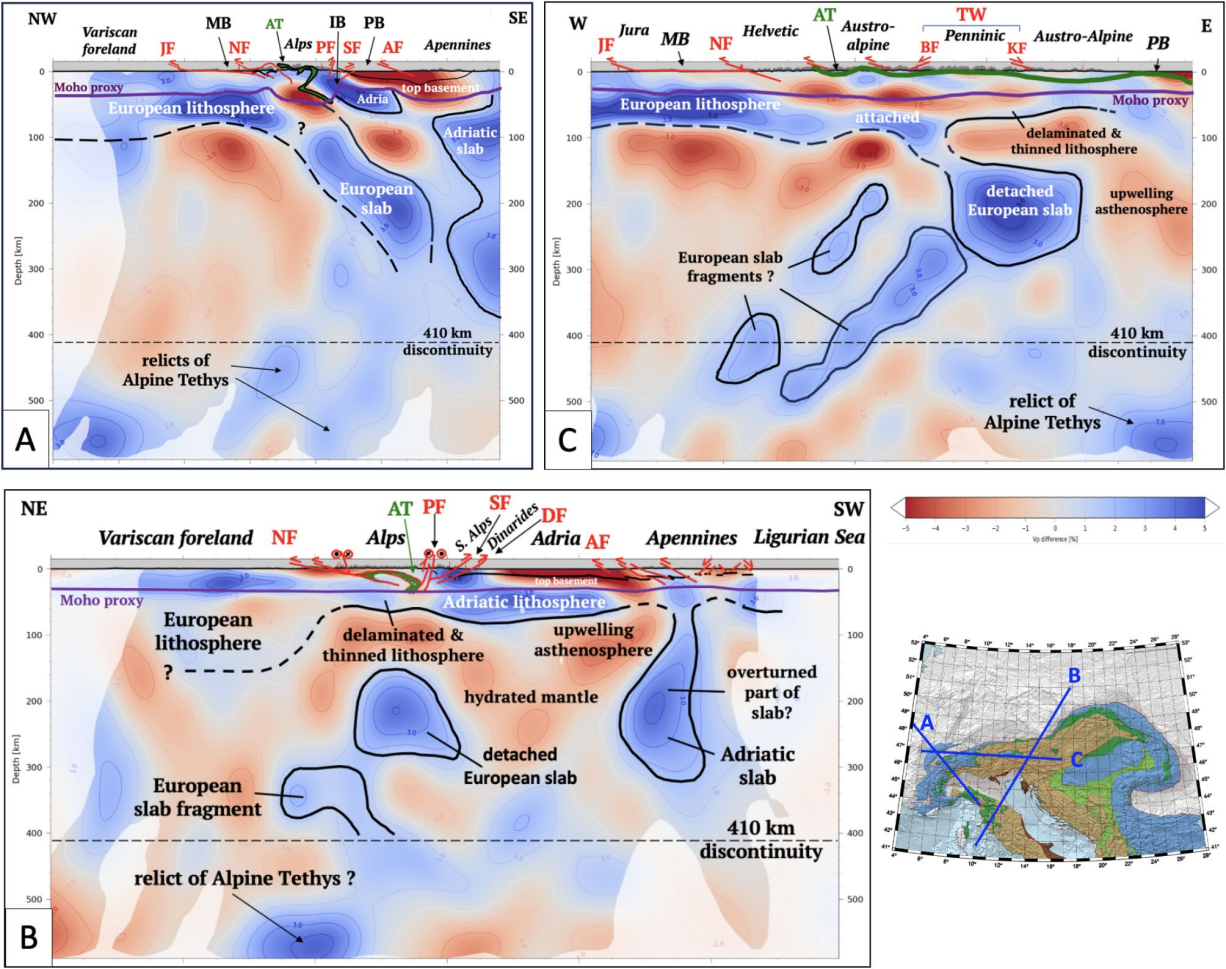
Teleseismic P-wave tomographic sections of the Central and Eastern Alps. Inset map shows traces of sections: (**A**) Positive P-wave slab anomaly interpreted as subducted European lithosphere still attached to the orogenic lithosphere of the Central Alps; (**B**) Positive P-wave anomaly interpreted as a remnant of European lithosphere subducted and detached since the onset of collision. The P-wave anomaly beneath the northern Apennines is subducted Adriatic lithosphere; (**C**) Orogen-parallel section highly oblique to dip of slab anomalies shows eastward detachment of European slab beneath the Tauern Window and lithospheric thinning towards the Pannonian Basin (modified from^19^). Tomography based on^34^ as interpreted by^29^ (their profiles 9, C and 15) and^19^. Abbreviations: AF = Apenninic Front, AT = Alpine Tethyan suture, BF = Brenner Normal Fault, DF = Dinaric Front. IV = Ivrea Geophysical Body, JF = Jura thrust front, KF = Katschberg Normal Fault, MB = Molasse foreland basin, NF = Northern orogenic front, PB = Pannonian Basin, SF = Southern orogenic front, PB = Po-Veneto Basin, PF = Periadriatic Fault, TW = Tauern Window.

**Fig. 4:**
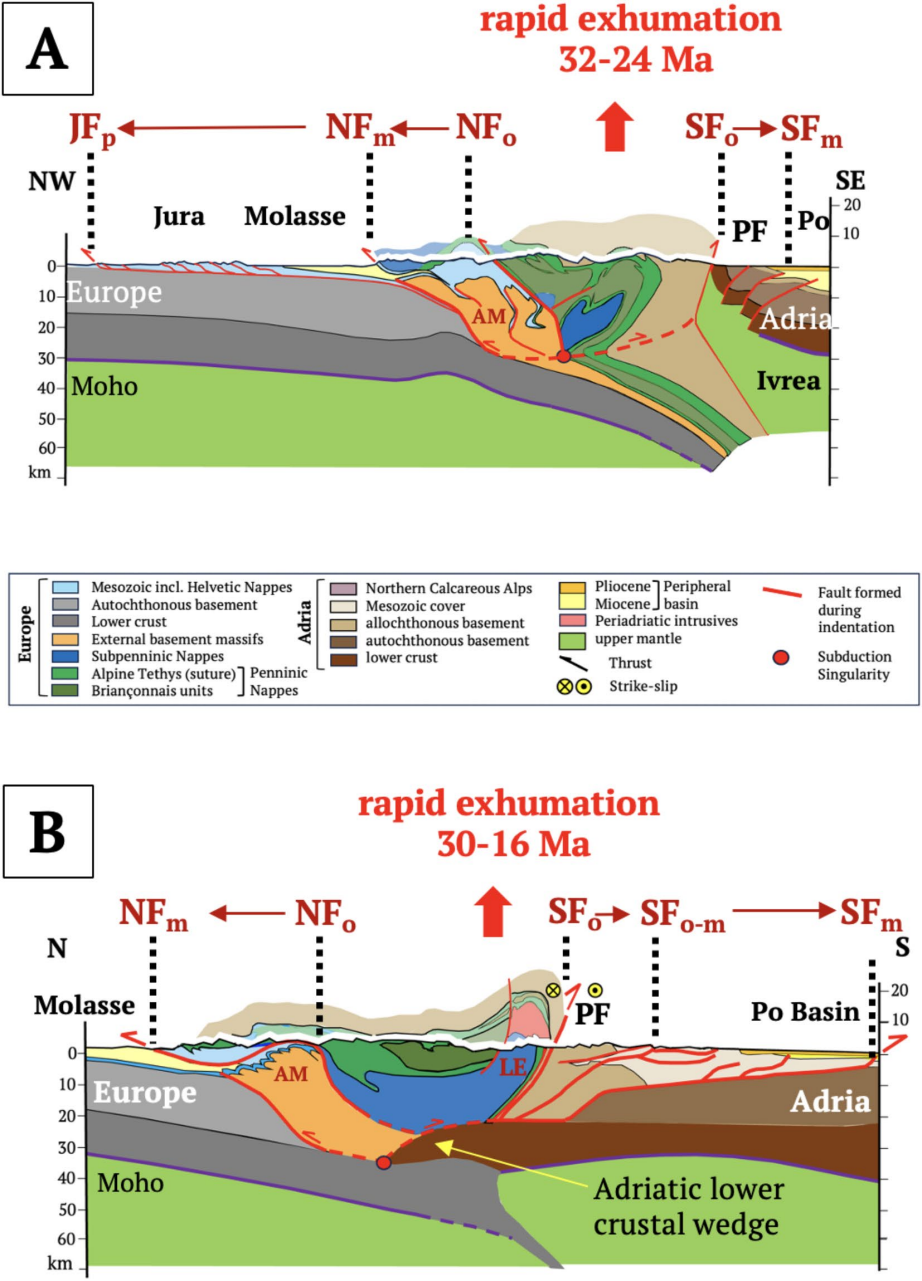
Central Alps: (**A**) NFP20W section of the western Central Alps showing Ivrea Body and advance of the orogenic fronts; (**B**) NFP20E section of the eastern Central Alps showing Adriatic lower crustal wedge and advance of orogenic fronts. Traces of cross sections in Fig. 2A. Red point—subduction singularity. Brown arrows and vertical dotted lines show migration of northern and southern orogenic fronts (NF, SF) from collision (Oligocene) to Present: Rapid exhumation rates (0.6–1 mm/a) and ages marked red in the retro-wedge from Fox et al.^35^. Cross section (**A**) modified from Schmid et al.^9^ and Burkhard & Sommaruga^36^ with deep structure based on controlled-source seismology^37^, local earthquake tomography^38^ and these methods combined with receiver functions^39^. Cross section (**B**) from Schmid et al.^15^ with deep structure partly based on controlled-source seismology in Pfiffner et al.^40^. Abbreviations: AM – Aar Massif, LE – Lepontine metamorphic dome, NF—Northern orogenic front, JF – Jura thrust front, SF – Southern orogenic front (subscripts: o – Oligocene, m – Miocene, p – Pliocene), PF – Periadriatic Fault.

Whereas the European slab beneath the Central Alps dips southeastward and may reach down to the Mantle Transition Zone (Fig. 3A), the slab beneath the Eastern Alps is mostly detached from the orogenic lithosphere at a depth of 60–150 km (Figs. 3B,C). The down-dip length of the detached slab anomaly is comparable with shortening estimates of Neogene structures in cross sections of the Eastern Alps, indicating that this anomaly comprises European continental lithosphere subducted since 23 Ma^19^. An Adriatic origin for this anomaly^27,28^ is unlikely given that its length far exceeds shortening of the Adriatic crust^29^, though a minor Adriatic contribution cannot be completely ruled out^30^. Slab anomalies at > 300km beneath both Central and Eastern Alps comprise mostly subducted oceanic lithosphere of Alpine Tethys^29^ based on the estimated pre-orogenic distance separating the European and Adriatic margins (≤ 440km, ^31^). Beneath the Central Alps, the slab probably includes subducted outliers of the European margin (e.g., Briançonnais, Fig. 4, ^32^). Exhumed relics of this subducted lithosphere are preserved in the ophiolitic nappes forming the Alpine Tethyan suture (dark green unit, Figs. 1, 4). Deep seismicity is restricted to the lower crust of the Central Alps^33^. The slab and slab segments are aseismic.

### Crustal structure

**Fig. 5:**
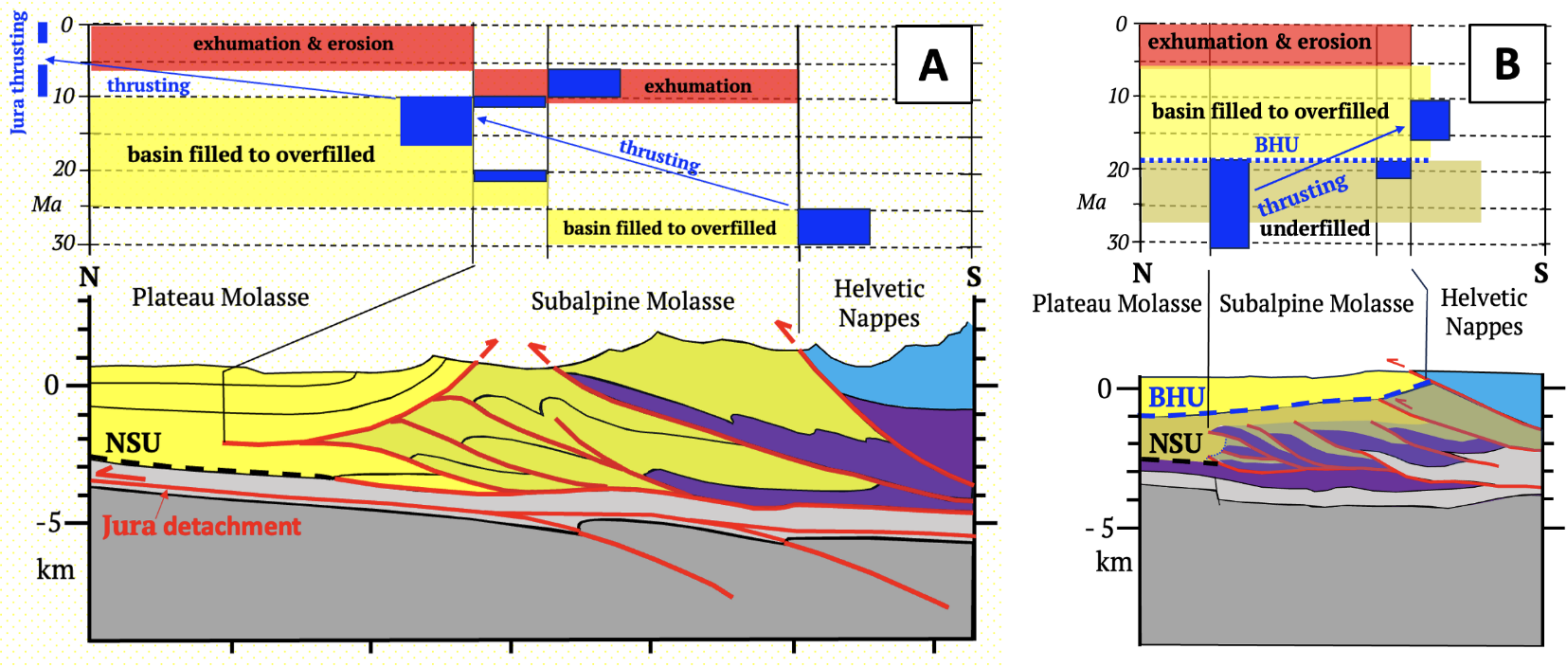
Cross sections of the northern orogenic front in the Central Alps (**A**) and Eastern Alps (**B**) modified, respectively, from von Hagke et al. (^16^, Entlebuch section) and Ortner et al. (^17^, Perwang section). Traces of sections in Fig. 2A. Symbols: Red = thrusts. NSU—Northern Slope Unconformity; BHU—Base Hall Unconformity, dashed blue line in (**B**), dark grey—European basement, light grey—Mesozoic cover, purple—Eocene flysch, yellow—filled to overfilled part of basin; light brown—in **B** underfilled part of basin below BHU; shaded area—Subalpine Molasse comprising mostly late Oligocene clastics. Age diagrams above sections show depositional ages (yellow and light brown, respectively, for filled/overfilled and underfilled parts of basin), thrusting ages (dark blue, as constrained by ages of youngest strata beneath the thrusts), and exhumation, uplift and erosion (red, constrained by thermochronology^16,43^). Note that in A, thrusting migrated northwards, i.e., towards the foreland, whereas in B it changed from north to south, stepping back into the orogen.

The Central Alps are asymmetrically structured, with a pro-wedge comprising north-directed thrusts and folds of European basement and detached cover (Figs. 4A,B, External Basement Massifs and Helvetic Nappes), and imbricated layers of the proximal foreland Molasse Basin (Fig. 5A). In the western Central Alps, a detachment bounding the pro-wedge extends northward beneath this basin to the Plio-Pleistocene Jura Front (Figs. 4A, 5A). Thrusts in the triangle zone along the proximal foreland basin were exhumed and reactivated at this time (Fig. 5A), concomitant with out-of-sequence thrusting and subvertical exhumation of the External Basement Massifs (AM in Figs. 4A,B). The retro-wedge comprises backfolded nappes and exhumed amphibolite-grade metamorphism in the Lepontine Dome^41^ intruded by late Oligocene syn-tectonic Periadriatic granitoids^42^. It is delimited by the steeply north-dipping Periadriatic Fault in front of rigid lower crustal and upper mantle rocks of the Ivrea Subindenter (Fig. 4A). In the eastern Central Alps, a sliver of Adriatic lower crust ~ 50 km long protrudes beneath the Oligo-Miocene retro-wedge (Fig. 4B). The southern thrust front bounding the Miocene retro-wedge is sealed by late Miocene-Pliocene sediments (Figs. 4A,B).

**Fig. 6:**
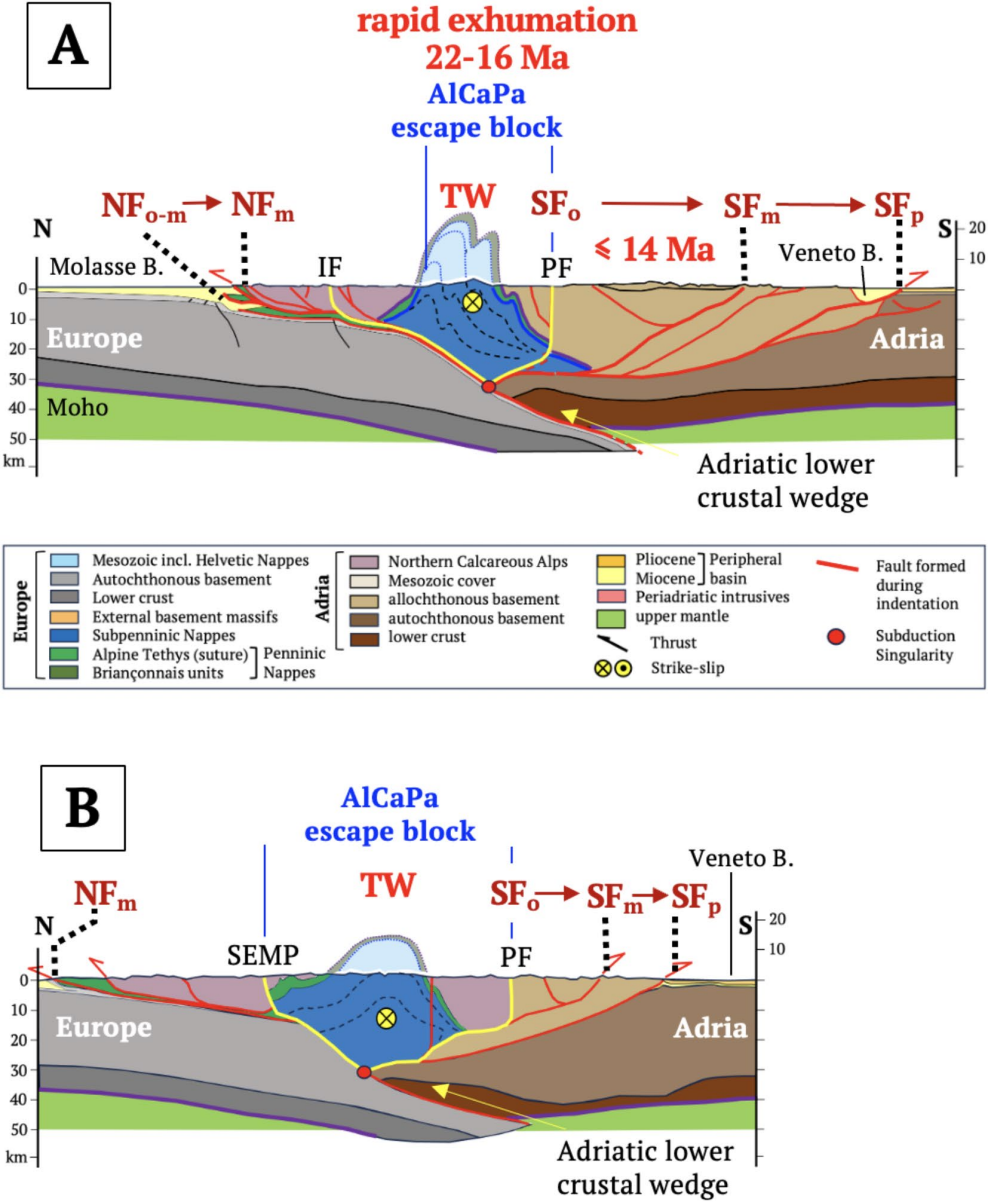
Eastern Alps: (**A**) TRANSALP section; (**B**) EASI section. Traces of sections in Fig. 2A. Red point at tip of lower crustal wedge – subduction singularity. Brown arrows and vertical dotted lines show migration of northern and southern orogenic fronts (NF, SF) from collision (Oligocene) to Present: Ages and rates of rapid exhumation (0.6–1.1 mm/a) and eastward motion of the AlCaPa escape block in the Tauern Window (TW) taken from Fox et al.^35^. Sections modified from^19^ based on controlled-source seismology^47–49^, receiver functions^50,51^ and local earthquake tomography^52^. Main structures: IF – Inntal Fault, NF and SF – Northern and Southern orogenic fronts, respectively (subscripts: o – Oligocene, m – Miocene, p – Pliocene), PF – Periadriatic Fault, Fault, SEMP – Salzach–Ennstal–Mariazell–Puchberg Fault; TW – Tauern Window. Colours as in legend to Fig. 4.

In contrast, the Eastern Alps are more symmetrical, with exhumed metamorphic units in their core (Tauern Window) and a pro-wedge including deformed late Cretaceous nappes of the Northern Calcareous Alps (Figs. 6A,B). The tip of the Oligo-Miocene pro-wedge extends ~ 10 km into the foreland basin and is cut by out-of-sequence, mid-late Miocene thrusts (Fig. 5B, ^44^). The retro-wedge comprises a long, mid-Miocene-to-Recent, south-vergent fold-and-thrust belt that is still active along the Po-Veneto hinterland basin (Figs. 2B, 5B). A sliver of Adriatic lower crust extends below and to the north of the Periadriatic Fault (Figs. 6A,B). Neogene upright folds and normal faults in the Tauern Window accommodated rapid exhumation and east-directed orogen-parallel extension in early to mid-Miocene time^45,46^. The Tauern Window and eastern Southern Alps directly overlie the detached part of the European slab (Fig. 3C).

The Alpine subduction singularity is presently located at the tip of the Adriatic lower crustal indenter (Figs. 4B, 5A) and, in the western Central Alps, at the detachment point between downgoing and backfolded units north of the Ivrea Subindenter (Fig. 5B). All of these points lie within the orogenic crust, indicating detachment at the interface between lower and intermediate crust. The European lower crust is thus part of the downgoing plate.

### Basins

**Fig. 7:**
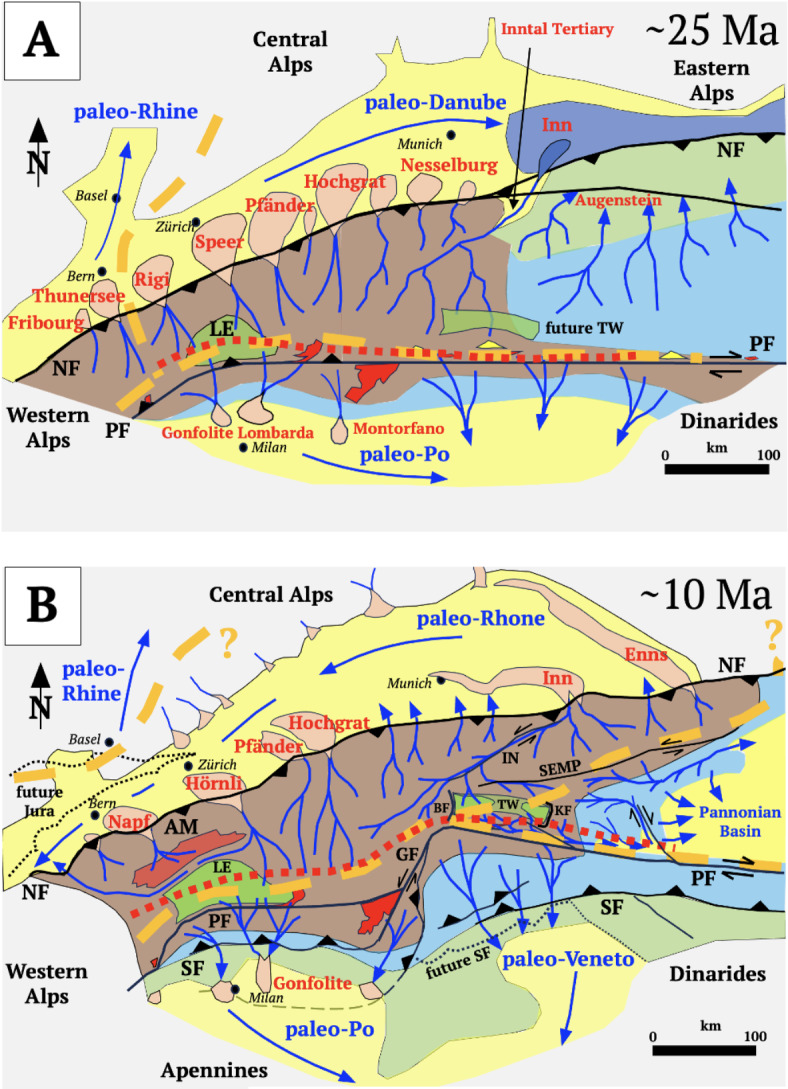
Paleogeography, drainage divides (orange dashed lines), inferred river flow (blue lines and arrows, rivers labelled) and trace of subduction singularity (red dotted line) in the Central and Eastern Alps in late Oligocene (**A**) and mid-Miocene times (**B**). Maps compiled from basinal, paleobathymetric and sedimentary provenance studies of^54,56,60–65^, and the morphometric reconstructions of Winterberg and Willet^66^. Morphology of sedimentary sinks: yellow – filled-to-overfilled basins with fluvial channels and floodplains,pink – alluvial megafans, fan deltas, coarse clastics (fan names labelled in red); blue – underfilled part of Eastern Alps foreland basin containing submarine fan (dark blue) and turbidites. Morphology of sedimentary source areas: brown—mountainous terrain (≤ 4000m a.sl); light blue – hilly terrain (≤ 2000m a.sl.); light green – lowlands including Augenstein deposits (see text). Geological features and symbols: AM – Aar Massif, BF – Brenner Normal Fault, GF – Giudicarie Fault, IN – Inntal Fault, KF—Katschberg Normal Fault, LE – Lepontine metamorphic dome (bright green), NF – Northern orogenic front, PF – Periadriatic Fault; SF – Southern orogenic front, SEMP—Salzachtal–Ennstal–Mariazell–Puchberg Fault, TW – Tauern Window with Tertiary metamorphic dome (bright green); bright red – exhuming Periadriatic intrusive bodies; yellow triangles – Periadriatic volcanics. Trace of the subduction singularity at the surface obtained by upward projection of singularity points from cross sections in Figs. 4 and 6 and from reconstructions of sections NFP20E^61^, their Fig. 2 for 20 Ma) and EASI^19^, their Fig. 9 for 14 and 23 Ma). Reference frame for motions between A and B is the European foreland.

The former shape and depth of the peripheral basins in Fig. 7 are constrained by sedimentary provenance studies, thermochronology and paleobathymetric estimates in basinal sediments as cited in the Fig. 5 caption. Onlap of alternating marine and near-shore sediments onto a south-dipping erosional slope unconformity in the foreland Molasse basin (NSU, Fig. 5) records the Oligocene advance of the Alpine pro-wedge. In the Central Alps after 30 Ma, erosion of both pro-and retro-wedges shed coarse clastics in prograding alluvial megafans and debris flows into the foreland^53^ and hinterland basins (Fig. 7A, Gonfolites; ^54^). However, whereas the Central Alpine part of the foreland basin was filled-to-overfilled^55^, the Eastern Alpine part remained underfilled, with hemipelagic and turbiditic sedimentation until ~ 19 Ma (Figs. 5A,B, 7B,^56^) when the basin experienced uplift and terrigenous infilling^57^ within only 1–2 Ma^58^. This dramatic event is marked by submarine erosion (BHU, Fig. 5B) that occurred during the aforementioned retreat of the northern thrust front^17^, as well as with a shift from eastward to westward axial drainage (Figs. 7A,B). Since 5 Ma, the foreland basin has undergone differential uplift and erosion of 0.6–2 km and 0.2–0.5 km, respectively, in its central and eastern parts^59^.

A curious feature of the Eastern Alps and the foreland basin is the late Oligocene embayment marked by clastics of the so-called Inntal Tertiary (Fig. 7A, ^60^). Eastward temporal correlatives of these sediments (Augenstein), originally deposited on hilly lowland terrain^67^, are presently found on karstified plateaus at ~ 1800m (Dachstein paleosurfaces) in the Northern Calcareous Alps. These paleosurfaces probably uplifted in early Late Miocene time (Fig. 7B, ^67^).

The Po-Veneto hinterland basin experienced subsidence after 25 Ma and accelerated subsidence from ~ 15 Ma onwards (Fig. 7B, ^62^) as indicated by the deposition of mid-late Miocene hemipelagic and turbiditic sediments^62,63^. Sandstone and coarse clastics originating from basement and Mesozoic cover rocks of the eastern Southern Alps and Austroalpine units mark the emergence of south-directed thrust sheets in the retrowedge^64^. Unlike the furrowed Molasse foreland basin, the Po-Veneto hinterland basin has a smooth surface and has not experienced appreciable uplift or erosion.

### Paleomorphology

Paleo-drainage divides between the Danube, Rhine, Rhône and Po River catchments in Fig. 7 are drawn schematically based on lithologies of source areas in the Alps found in the peripheral basins (references in caption). From ~ 25 Ma onward, alluvial fans in proximal parts of the central Molasse contain detritus from the highest basement nappes (Austroalpine), while the Gonfolites in the western Southern Alps include components of Periadriatic granite and Lepontine metamorphics^68^. This indicates that the Oligocene Danube-Po drainage divide in the Central Alps ran just north of the Periadriatic Fault (Fig. 7A, ^61^). The influx of low-grade metamorphic detritus in the Central Alpine Molasse after ~ 20 Ma indicates the erosion of more external parts of the pro-wedge. Rivers flowed away from the exhumed Lepontine Dome and around the External Basement Massifs, suggesting that the Po-Rhône drainage divide had migrated northwards with respect to the foreland (Fig. 7B). Stable isotope studies indicate that altitudes of source areas for the clastic alluvial fans equaled or even exceeded modern altitudes by early-mid Miocene time (≥ 4000m a.sl., ^69^).

In the Eastern Alps, the Oligo-to-Miocene Po-Danube drainage divide also migrated northwards, away from the Periadriatic Fault and magmatites (Fig. 7A,B) as indicated in the eastern Molasse basin by the cessation of Periadriatic detrital input and increased metamorphic detritus derived from the exhuming units in the Tauern Window^60^. Beginning in early middle Miocene time detritus from these sources was also deposited eastwards into the Danube catchment of the proto-Pannonian Basin^65^. This supports the notion that the drainage divide bifurcated between 20–15 Ma, forming separate Po-Danube and Rhône-Danube divides that diverged eastward from the Tauern Window area (Fig. 7B). Drainage was channeled into valleys along Neogene oblique-slip faults and pull-apart basins accommodating orogen-parallel escape toward the Pannonian Basin^60^.

In the Central and Eastern Alps, the main drainage divide generally followed the trace of the subduction singularity projected to the surface (Fig. 7) for Oligocene and Miocene time slices. East of the Tauern Window, this relationship is unclear given the paucity of high-resolution seismic data.

## Discussion

**Fig. 8:**
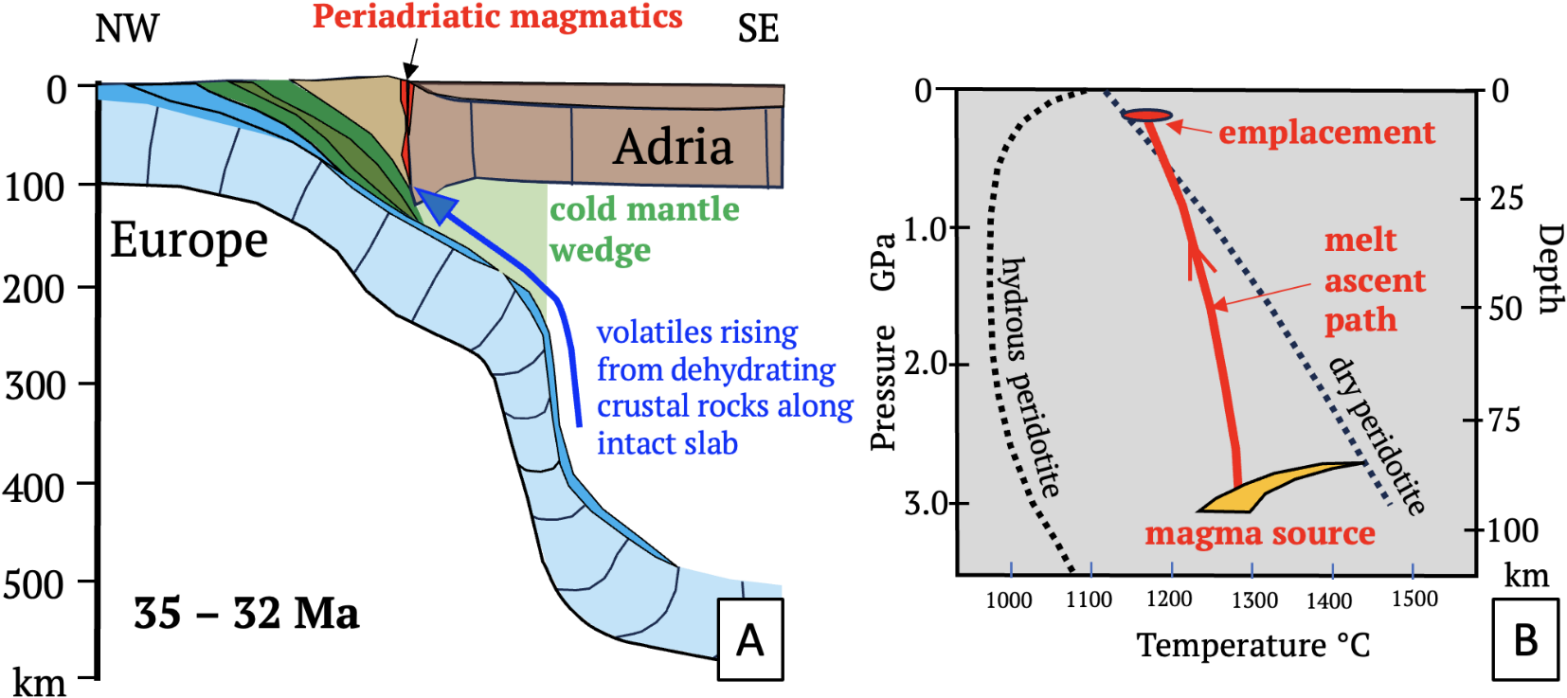
Slab configuration and Tertiary magmatism in the Alps: (**A**) cross section of the nascent Alps during Adria-Europe collision (modified from^32^) showing proposed path of fluids (blue arrow) from dehydrating sediments along the still-intact European slab through the cold mantle wedge to the base of the orogenic wedge^70^. Periadriatic magmatic rocks (red) emplaced along the Periadriatic Fault, (**B**) conditions of melt ascent and emplacement (thick red line with arrow, simplified from^70^). Colours in A: light blue – subducting European lithospheric mantle, blue – European crust; green – Penninic units (include Alpine Tethys and Briançonnais); light brown – accreted Adriatic crust; darker brown – Adriatic lithosphere.

**Fig. 9:**
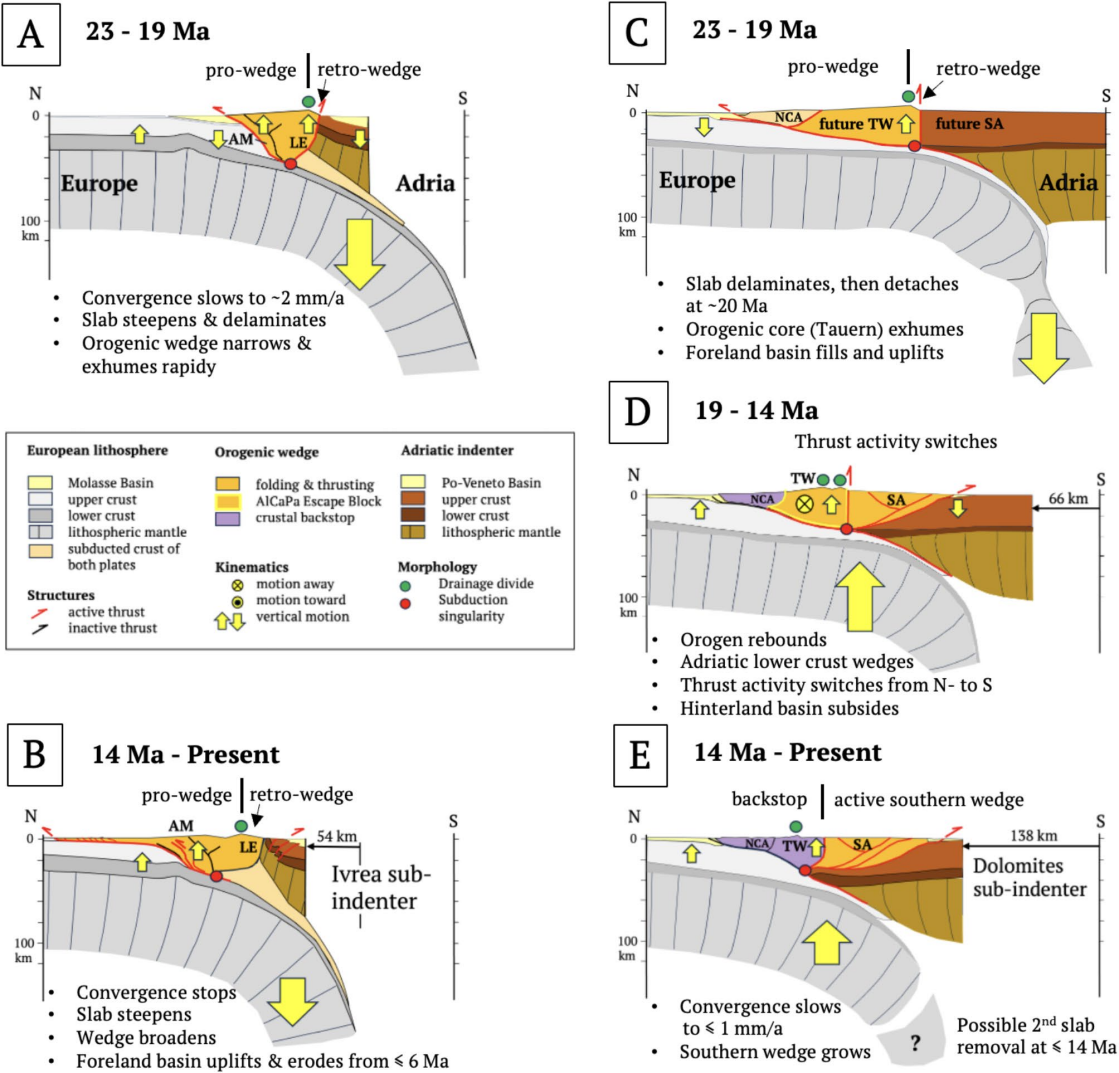
Neogene time slices of the western Central (**A**, **B**) and Eastern Alps (**C**, **D**, **E**), respectively, along NFP20W and TRANSALP profiles (lines 4A and 6A in Fig. 2A). Slices show migration of subduction singularities (red circles) and main drainage divides (green circles) during Adriatic indentation with respect to Europe. Yellow arrows indicate vertical motions due primarily to subsurface forces acting on the orogenic wedge. Arrow size indicates magnitude. Post-23 Ma shortening estimates taken from McPhee & Handy^19^ (138 km), Schmid et al.^9^ and Burkhard & Sommaruga^36^ (54 km). Slab lengths are not to scale and are longer than depicted here. AM - Aar Massif, LE - Lepontine metamorphic dome, NCA - late Cretaceous nappes of Northern Calcareous Alps, SA - Southern Alps, TW - metamorphic core of Tauern Window.

Cross sections from collision (Fig. 8) to indentation in the Central and Eastern Alps (Fig. 9) depict motions of the subduction singularity, thrust fronts and drainage divides with respect to the European Plate. Shortening and convergence in these sections come from 2D- and 3D-section balancing referenced in the figure captions. Slabs in Fig. 9 are not drawn to scale.

During collision (Fig. 8), Adria-Europe convergence at 6–7.4 mm/a^31,71^ was accommodated in the Eastern and Central Alps along a common active margin^32^. In the Central Alps, the slab steepened but never detached (Figs. 3A, 8A, 9A), which precludes slab breakoff as an explanation for Oligocene Periadriatic magmatism^72,73^. Instead, we follow Müntener et al.^70^ in attributing this magmatism to decompression melting in the rapidly exhuming retro-wedge fed by K-rich fluids that fluxed the cold mantle wedge on their way up from subducted sediments along the slab (Fig. 8A,B). Note that slab steepening is similar to “rollback orogeny” of Kissling & Schlunegger^74^. However, unlike “classical” rollback where upper-plate extension accommodates subduction relative to a mantle reference frame (e.g., ^5^), slab steepening beneath the Neogene Alps occurred with little, if any, plate convergence and no upper-plate extension or magmatism^32^.

With the onset of Adriatic indentation at ~ 32 Ma, the convergence rate dropped to ~ 2 mm/a^32^ and, after 20 Ma, to ~ 0.5 mm/a^6^. Growth of the Central Alpine orogenic wedge during slab steepening in the absence of appreciable plate convergence is expected to have increased wedge buoyancy relative to the downward pull of the slab (Fig. 9A). The wedge taper increased until shear stresses exceeded the shear strength at the wedge base. The resultant “pop-up” and spreading of the entire wedge not only exhumed the Lepontine metamorphics, but increased topography and erosion, leading to the massive influx of clastic sediments observed along the foreland basin (Fig. 7A). With continued indentation, the subduction singularity migrated away from the Ivrea Subindenter (Fig. 9B), triggering further Mio-Pliocene exhumation of the External Basement Massifs while the pro- and retro-wedges expanded northwards (Jura Mountains) and southwards (Po-Veneto Basin).

In the Eastern Alps, the slab steepened and delaminated further northwards than in the Central Alps (Fig. 9C), conceivably because its downward pull was greater without subducted Briançonnais continental lithosphere^29^. Transfer of this pull to the foreland provides a viable mechanism for prolonged subsidence of the eastern, underfilled part of the foreland basin that slightly outlasted retreat of pro-wedge thrusting after ~ 19 Ma. Northward migration of the subduction singularity facilitated indentation, in turn inducing Tauern exhumation and orogen-parallel escape, and shifting thrust activity from north to south (Fig. 9D). The Northern Calcareous Alps (NCA) became the crustal backstop to the Neogene wedge comprising the warm, freshly exhumed nappes of the Tauern Dome. The Po-Danube drainage divide, which had hitherto tracked the subduction singularity northward, bifurcated above the exhuming and eastwardly extruding AlCaPa escape block (Fig. 9B). Slab detachment at ~ 20 Ma then triggered rapid infill and uplift of the eastern foreland basin^29,75^, and indeed, uplift of the entire Eastern Alps including the Dachstein paleosurfaces of the NCA with their Oligocene Augenstein clastic cover^24^. By ~ 14 Ma, the Tauern nappes had cooled to < 300°C^76^ and hardened, thus becoming part of the crustal backstop. Continued indentation (≤ 14 Ma, ^19^) involved northward and upward migration of the subduction singularity at the tip of the indenting Adriatic lower crustal wedge, shifting deformation into the Dolomites Subindenter and lengthening the orogenic wedge southwards (Fig. 9E). Nappe stacking in the Southern Alps, possibly augmented by hinterland weakening^77^, accelerated subsidence of the Po-Veneto Basin. The new thrust wedge in the eastern Southern Alps is still growing today, even as Adria-Europe convergence has slowed to ≤ 1 mm/a^78^.

Slab steepening, detachment and lower crustal wedging are key processes shaping mountain belts during the late stages of collision. Whereas the primary effect of slab steepening is to shift subsurface loading towards the foreland, slab detachment decreases loading, leading to uplift and erosion^79^. An equally potent effect of slab detachment is to change the taper angle of the orogenic wedge, thus focussing or defocussing exhumation and denudation, depending on the subsurface boundary conditions. Indentation, particularly lower crustal wedging, also causes subduction singularities and drainage divides to migrate toward the foreland, even in the absence of slab detachment, as in the Central Alps. However, drastic along-strike changes as in the Eastern Alps occur where slab retreat and detachment combined with Adriatic indentation facilitated orogen-parallel extrusion and lithospheric thinning in the upper plate of the retreating Carpathian subduction^4^.

Collision zones with along-strike changes in structure abound the world over, suggesting that similar late-orogenic processes effected their arcuation and segmentation. For example, the switch in subduction polarity beneath Taiwan involves tearing and steepening of the downgoing Eurasian Plate (e.g., ^80^), together with an orogen-parallel shift of the intracrustal subduction singularity to the retreating boundary of the delaminating Philippine Sea Plate(^81^, their Fig. 13). The surface manifestation of this shift is pronounced topography and uplift of northern Taiwan juxtaposed with dramatic subsidence of the adjacent Okinawa Sea back-arc basin. Similarly, the Himalayan–Tibetan orogenic system that accommodated some 1500 km of India-Eurasia collisional convergence^82^ is underlain by laterally discontinuous slab anomalies, particularly in the 100–400 km depth interval^83^. This has been interpreted as evidence for orogen-parallel detachment of the Indian slab since ~ 25–10 Ma and linked to northward movement of the Indian Plate and Himalayan orogen over the overturned and sinking slab fragment^82^. This in turn necessitates the migration of subduction singularities, both at the base of and within the orogenic lithosphere, that are potentially linked to along-strike variations in topography^84^ and uplift rate^85^. Higher density seismic arrays combined with tectonic, thermochronologic and surface studies may shed better light on the key role of the lower crust in accommodating such dramatic changes.

## Data Availability

Data availability statement—The datasets used and/or analysed during the current study are available from the corresponding author on reasonable request. Papers cited here that are related to the project Mountain-Building in Four Dimensions are freely available at http://www.sppmountainbuilding.de/publications/index.html. These papers include links to data repositories in the pertinent journals and research institutions. Results in the section on Mantle Structure were obtained from published data of the temporary AlpArray Seismic Network (2015) under FDSN network code Z3 (Hetényi et al. 2018, http://alparray.ethz.ch/en/seismic_network/backbone/data-policy-and-citation/).

## References

[1] Hetényi, G. et al. The AlpArray Seismic Network: A Large-Scale European Experiment to Image the Alpine Orogen. *Surv. Geophys.*10.1007/s10712-018-9472-4 (2018). 10.1007/s10712-018-9472-4 PMC6428228 30956376

[2] Schmid, S. M., Fügenschuh, B., Kissling, E. & Schuster, R. Tectonic map and overall architecture of the Alpine orogen. *Eclog. Geol. Helv.* **97**, 93–117. 10.1007/s00015-004-1113-x (2004).

[3] Schmid, S. M. et al. The Alpine-Carpathian-Dinaridic orogenic system: Correlation and evolution of tectonic units. *Swiss J. Geosci.* **101**, 139–183. 10.1007/s00015-008-1247-3 (2008).

[4] Horváth, F. et al. Formation and deformation of the Pannonian Basin: Constraints from observational data. *Geol. Soc. Mem.* **32**, 191–206. 10.1144/GSL.MEM.2006.032.01.11 (2006).

[5] Royden, L. H. & Burchfiel, B. C. Are systematic variations in thrust belt style related to plate boundary processes? (The western Alps versus the Carpathians). *Tectonics* **8**, 51–61. 10.1029/TC008i001p00051 (1989).

[6] Le Breton, E., Handy, M. R., Molli, G. & Ustaszewski, K. Post-20 Ma motion of the Adriatic plate: New constraints from surrounding orogens and implications for crust-mantle decoupling. *Tectonics* **36**, 202–217. 10.1002/2016TC004443 (2017).

[7] D’Agostino, N. et al. Active tectonics of the Adriatic region from GPS and earthquake slip vectors. *J. Geophys. Res.* **113**, B12413. 10.1029/2008JB005860 (2008).

[8] Ustaszewski, K. et al. A map-view restoration of the Alpine–Carpathian–Dinaridic system for the Early Miocene. *Swiss J. Geosci.* **101**, 273–294. 10.1007/s00015-008-1288-7 (2008).

[9] Schmid, S.M. et al. G. Ivrea mantle wedge, arc of the Western Alps, and kinematic evolution of the Alps–Apennines orogenic system. *Swiss J Geosci* (2017). 10.1007/s00015-016-0237-0

[10] Vignaroli, G. et al. Subduction polarity reversal at the junction between the Western Alps and the Northern Apennines. *Italy. Tectonophysics* **450**, 34–50. 10.1016/j.tecto.2007.12.012 (2008).

[11] Brunsmann, Q., Rosenberg, C. L. & Bellahsen, N. The Western Alpine arc: a review and new kinematic model. *Comptes Rendus Géoscience* **356**, 231–263. 10.5802/crgeos.253 (2024).

[12] Király, Á., Faccenna, C. & Funiciello, F. Subduction zones interaction around the Adria microplate and the origin of the Apenninic arc. *Tectonics* **37**, 3941–3953. 10.1029/2018TC005211 (2018).

[13] Molli, G. et al. Geology of the Western Alps-Northern Apennine junction area – A regional review. *J. Virtual Exp.* **36**, 1–49. 10.3809/jvirtex.2010.00215 (2010).

[14] Roure, F. et al. The ECORS-CROP Alpine seismic traverse. *Mémoires Société Géologique de France* **170**, 113 (1996).

[15] Schmid, S. M. et al. Geophysical-geological transect and tectonic evolution of the Swiss-Italian Alps. *Tectonics* **15**, 1036–1064. 10.1029/96TC00433 (1996).

[16] von Hagke, C. et al. Linking the northern Alps with their foreland: The latest exhumation history resolved by low-temperature thermochronology. *Tectonics*10.1029/2011TC003078 (2012).

[17] Ortner, H.S. et al. Geometry, amount, and sequence of thrusting in the Subalpine Molasse of western Austria and southern Germany, European Alps. *Tectonics*10.1002/2014TC003550 (2015).

[18] Gebrande, H. et al. First deep seismic reflection images of the Eastern Alps reveal giant crustal wedges and transcrustal ramps. *Geophys. Res. Lett.*10.1029/2002GL014911 (2002).

[19] McPhee, P., Handy, M.R. Post-collisional reorganization of the Eastern Alps in 4D – Crust and mantle structure. 10.1029/2024TC008374. (2024).

[20] Midtkandal, I., Brun, J.-P., Gabrielsen, R. H. & Huismans, R. S. Control of lithosphere rheology on subduction polarity at initiation: Insights from 3D analogue modelling. *Earth Planet. Sci. Lett.* **361**, 219–228. 10.1016/j.epsl.2012.10.026 (2012).

[21] Dando, B. D. E. et al. Teleseismic tomography of the mantle in the Carpathian-Pannonian region of central Europe. *Geophys. J. Int.* **186**, 11–31. 10.1111/j.1365-246X.2011.04998.x (2011).

[22] Wortel, M. J. R. & Spakman, W. Subduction and slab detachment in the Mediterranean-Carpathian region. *Science* **290**, 1910. 10.1126/science.290.5498.1910 (2000). 11110653 10.1126/science.290.5498.1910

[23] Kissling, E. et al. Lithosphere structure and tectonic evolution of the Alpine arc: new evidence from high-resolution teleseismic tomography. *In Eur. Lith. Dyn.* **32**, 129–145. 10.1144/GSL.MEM.2006.032.01.08 (2006).

[24] Handy, M. R. et al. Reconstructing the Alps–Carpathians–Dinarides as a key to understanding switches in subduction polarity, slab gaps, and surface motion. *Int. J. Earth Sci.* **104**, 1–26. 10.1007/s00531-014-1060-3 (2015).

[25] Beaumont, C., Fullsack, P. & Hamilton, J. Styles of crustal deformation in compressional orogens caused by subduction of the underlying lithosphere. *Tectonophysics* **232**, 119–132. 10.1016/0040-1951(94)90079-5 (1994).

[26] Naylor, M. & Sinclair, H. D. Pro- vs. retro-foreland basins. *Basin Res.* **20**, 285–303. 10.1111/j.1365-2117.2008.00366.x (2008).

[27] Lippitsch, R., Kissling, E. & Ansorge, J. Upper mantle structure beneath the Alpine orogen from high-resolution teleseismic tomography. *J. Geophys. Res.* **108**, 2376. 10.1029/2002JB002016 (2003).

[28] Karousová, H. et al. Upper-mantle structure beneath the southern Bohemian Massif and its surroundings imaged by high-resolution tomography. *Geophys. J. Int.* **194**, 1203–1215. 10.1093/gji/ggt159 (2013).

[29] Handy, M. R. et al. Orogenic lithosphere and slabs in the greater Alpine area - Interpretations based on teleseismic P-wave tomography. *Solid Earth* **12**, 2633–2669. 10.5194/se-12-2633-2021 (2021).

[30] Kästle, E. D. et al. Slab break-offs in the Alpine subduction zone. *Int. J. Earth Sci.*10.1007/s00531-020-01821-z (2020).

[31] Le Breton, E. et al. Kinematics and extent of the Piemont-Liguria Basin – implications for subduction processes in the Alps. *Solid Earth* **12**, 885–913. 10.5194/se-12-885-2021 (2021).

[32] Handy, M. R. et al. Reconciling plate-tectonic reconstructions with the geological-geophysical record of spreading and subduction in the Alps. *Earth Sci. Rev.* **102**, 121–158. 10.1016/j.earscirev.2010.06.002 (2010).

[33] Singer, J. et al. Alpine lithosphere slab rollback causing lower crustal seismicity in northern foreland. *Earth Planet. Sci. Lett.* **397**, 42–56. 10.1016/j.epsl.2014.04.002 (2014).

[34] Paffrath, M., Friederich, W., Schmid, S.M., Handy, M.R., and the AlpArray and AlpArray-Swath D Working Groups. Imaging structure and geometry of slabs in the greater Alpine area – a P-wave travel-time tomography using AlpArray Seismic Network data. *Solid Earth*. 10.5194/se-12-2671-2021, (2021).

[35] Fox, M. et al. The exhumation history of the European Alps inferred from linear inversion of thermochronometric data. *Am. J. Sci.* **316**, 505–541. 10.2475/06.2016.01 (2016).

[36] Burkhard, M., Sommaruga, A. Evolution of the western Swiss Molasse basin: structural relations with the Alps and the Jura belt. In: Mascle, A. et al., Cenozoic Foreland Basins of Western Europe. *Geol. Soc. Spec. Publ.* **134**, 279–298 10.1144/GSL.SP.1998.134.01.13, (1998).

[37] Pfiffner, O.A. et al. Deep Structure of the Swiss Alps: Results of NRP 20. Birkhäuser, Basel (1997).

[38] Diehl, T., Husen, S., Kissling, E. & Deichmann, N. High‐resolution 3‐D P‐wave model of the Alpine crust. *Geophys. J. Int.* **179**(2), 1133–1147. 10.1111/j.1365-246X.2009.04331.x (2009).

[39] Spada, M. et al. Combining controlled-source seismology and receiver function information to derive 3-D Moho topography for Italy. *Geophys. J. Int.* **194**, 1050–1068. 10.1093/gji/ggt148 (2013).

[40] Pfiffner, O. A. et al. Crustal shortening in the Alpine Orogen: Results from deep seismic reflection profiling in the eastern Swiss Alps, Line NFP 20-east. *Tectonics* **9**, 1327–1355. 10.1029/TC009i006p01327 (1990).

[41] Berger, A. et al. The relation between peak metamorphic temperatures and subsequent cooling during continent–continent collision (western Central Alps, Switzerland). *Swiss J. Geosci.* **113**, 4. 10.1186/s00015-020-00356-4 (2020).

[42] Rosenberg, C. L. Shear zones and magma ascent: A model based on a review of the Tertiary magmatism in the Alps. *Tectonics*10.1029/2003TC001526 (2004).

[43] Mock, S. et al. Long-wavelength late-Miocene thrusting in the north Alpine foreland: Implications for late orogenic processes. *Solid Earth* **11**, 1823–1847. 10.5194/se-11-1823-2020 (2020).

[44] Ortner, H. et al. The northern Deformation Front of the European Alps. In *Geodynamics of the Alps 3* (Bellahsen, N., Rosenberg, C., Eds.), ISTE-Wiley, London (2023).

[45] Rosenberg, C. L. et al. Relating collisional kinematics to exhumation processes in the Eastern Alps. *Earth-Sci. Rev.* **176**, 311–344. 10.1016/j.earscirev.2017.10.013 (2018).

[46] Scharf, A. et al. Modes of orogen-parallel stretching and extensional exhumation in response to microplate indentation and roll-back subduction (Tauern Window, Eastern Alps). *Int. J. Earth Sci.* **102**, 1627–1654. 10.1007/s00531-013-0894-4 (2013).

[47] Castellarin, A., Vai, G. B. & Cantelli, L. The Alpine evolution of the Southern Alps around the Giudicarie faults: A Late Cretaceous to Early Eocene transfer zone. *Tectonophysics* **414**, 203–223. 10.1016/j.tecto.2005.10.019 (2006).

[48] Lüschen, E. et al. Orogenic structure of the Eastern Alps, Europe, from TRANSALP deep seismic reflection profiling. *Tectonophysics* **388**, 85–102. 10.1016/j.tecto.2004.07.024 (2004).

[49] Castellarin, A. & Cantelli, L. Neo-Alpine evolution of the Southern Eastern Alps. *J. Geodyn.* **30**, 251–274. 10.1016/S0264-3707(99)00036-8 (2000).

[50] Kummerow, J. et al. A natural and controlled source seismic profile through the Eastern Alps: TRANSALP. *Earth Planet. Sci. Lett.* **225**, 115–129. 10.1016/j.epsl.2004.05.040 (2004).

[51] Mroczek, S. et al. the SWATH-D Working Group, the AlpArray Working Group. Investigating the Eastern Alpine–Dinaric transition with teleseismic receiver functions: Evidence for subducted European crust. *Earth Planet. Sci. Lett.*10.1016/j.epsl.2023.118096 (2023).

[52] Jozi Najafabadi, A. et al. Crustal and upper mantle structure of the Southern and Eastern Alps based on high-resolution 3-D P- and S-wave models from local earthquake tomography. *Solid Earth*10.1029/2021JB0231 (2022).

[53] Schlunegger, F. & Castelltort, S. Immediate and delayed signal of slab breakoff in Oligo/Miocene Molasse deposits from the European Alps. *Nat. Commun. Sci. Rep.* **6**, 31010. 10.1038/srep31010 (2016). 10.1038/srep31010 PMC4980627 27510939

[54] Sciunnach, D. et al. The Monte Orfano Conglomerate revisited: Stratigraphic constraints on Cenozoic tectonic uplift of the Southern Alps (Lombardy, northern Italy). *Int. J. Earth Sci.* **99**, 1335–1355. 10.1007/s00531-009-0452-2 (2010).

[55] Sinclair, H. D. & Allen, P. A. Vertical versus horizontal motions in the Alpine orogenic wedge: Stratigraphic response in the foreland basin. *Basin Res.* **4**, 215–232. 10.1111/j.1365-2117.1992.tb00046.x (1992).

[56] Kuhlemann, J. & Kempf, O. Post-Eocene evolution of the North Alpine foreland basin and its response to Alpine tectonics. *Sediment. Geol.* **152**, 45–78. 10.1016/S0037-0738(02)00099-2 (2002).

[57] Le Breton, E. et al. et al. Early Miocene tectono-sedimentary shift in the eastern north Alpine foreland basin and its relation to changes in tectonic style in the eastern Alps. *Abstracts of the Annual AlpArray and 4DMB Scientific Meeting*, Bad Hofgastein, Austria, 10.17169/REFUBIUM-41056. (2023).

[58] Hülscher, J. et al. Selective Recording of Tectonic Forcings in an Oligocene/Miocene Submarine Channel System: Insights From New Age Constraints and Sediment Volumes From the Austrian Northern Alpine Foreland Basin. *Front. Earth Sci.*10.3389/feart.2019.00302 (2019).

[59] Baran, R., Friedrich, A. M. & Schlunegger, F. The late Miocene to Holocene erosion pattern of the Alpine foreland basin reflects Eurasian slab unloading beneath the western Alps rather than global climate change. *Lithosphere* **6**, 124–131. 10.1130/L307.1 (2014).

[60] Frisch, W. et al. Palinspastic reconstruction and topographic evolution of the Eastern Alps during late Tertiary tectonic extrusion. *Tectonophysics* **297**, 1–15. 10.1016/S0040-1951(98)00160-7 (1998).

[61] Schlunegger, F., Melzer, J. & Tucker, G. E. Climate, exposed source-rock lithologies, crustal uplift and surface erosion: a theoretical analysis calibrated with data from the Alps/Northern Alpine Foreland Basin system. *Int. J. Earth Sci.* **90**, 484–499. 10.1007/s005310100174 (2001).

[62] Mancin, N. et al. The Friulian-Venetian Basin II: Paleogeographic evolution and subsidence analysis from micropaleontological constraints. *Ital. J. Geosci.* **135**, 460–473. 10.3301/IJG.2015.34 (2016).

[63] Mellere, D., Stefani, C. & Angevine, C. Polyphase tectonics through subsidence analysis: The Oligo-Miocene Venetian and Friuli Basin, north-east Italy. *Basin Res.* **12**, 159–182. 10.1046/j.1365-2117.2000.00120.x (2000).

[64] Stefani, C. et al. Provenance and paleogeographic evolution in a multi-source foreland: The Cenozoic Venetian-Friulian Basin (NE Italy). *J. Sediment. Res.* **77**, 867–887. 10.2110/jsr.2007.083 (2007).

[65] Rybár, S. et al. Biostratigraphy, sedimentology and paleoenvironments of the northern Danube Basin: Ratkovce 1 well case study. *Geologica Carpathica* **66**, 51–67. 10.1515/geoca-2015-0010 (2015).

[66] Winterberg, S. & Willet, S. D. Greater Alpine river network evolution: Interpretations based on novel drainage analysis. *Swiss J. Geosci.* **112**, 3–22. 10.1007/s00015-018-0332-5 (2019). 32214983 10.1007/s00015-018-0332-5 PMC7081830

[67] Frisch, W. et al. The Dachstein paleosurface and the Augenstein Formation in the Northern Calcareous Alps – a mosaic stone in the geomorphological evolution of the Eastern Alps. *Int. J. Earth Sci.* **90**, 500–518. 10.1007/s005310000189 (2001).

[68] Bernoulli, D., Giger, M. & Müller, D. W. Sr-isotope stratigraphy of the Gonfolite Lombarda Group (South-Alpine Molasse, northern Italy) and radiometric constraints for its age of deposition. *Eclogae Geol. Helv.* **86**, 751–767 (1993).

[69] Ballian A. et al. Stable isotope paleoaltimetry reveals Early to Middle Miocene along-strike elevation differences of the European Alps. 10.5194/egusphere-egu24-18901, (2024).

[70] Müntener, O., Ulmer, P. & Blundy, J. Superhydrous arc magmas in the Alpine context. *Shedding Light Eur. Alps Elements* **17**, 35–40. 10.2138/gselements.17.1.35 (2021).

[71] Van Hinsbergen, D. J. J. et al. Orogenic architecture of the Mediterranean region and kinematic reconstruction of its tectonic evolution since the Triassic. *Gondwana Res.* **81**, 79–229. 10.1016/j.gr.2019.07.009 (2020).

[72] Dietrich, V. J. Evolution of the Eastern Alps – A plate tectonics working hypothesis. *Geology* **4**, 147–152. 10.1130/0091-7613(1976)4%3c147:EOTEAA%3e2.0.CO;2 (1976).

[73] von Blanckenburg, F. & Davies, J. H. Slab breakoff: A model for syn-collision magmatism and tectonics in the Alps. *Tectonics* **14**, 120–131. 10.1029/94TC02051 (1995).

[74] Kissling, E. & Schlunegger, F. Rollback Orogeny Model for the Evolution of the Swiss Alps. *Tectonics* **37**, 1097–1115. 10.1002/2017TC004762 (2018).

[75] Schlunegger, F. & Kissling, E. Slab load controls beneath the Alps on the source-to-sink sedimentary pathways in the Molasse Basin. *Geosciences* **12**, 226. 10.3390/geosciences12060226 (2022).

[76] Bertrand, A. et al. Exhumation mechanisms of the Tauern Window (Eastern Alps) inferred from apatite and zircon fission track thermochronology. *Tectonics* **36**, 207–228. 10.1002/2016TC004133 (2017).

[77] Bertotti, G., Picotti, V. & Cloetingh, S. Lithospheric weakening during “retroforeland” basin formation: Tectonic evolution of the central South Alpine foredeep. *Tectonics* **17**, 131–142. 10.1029/97TC02066 (1998).

[78] Serpelloni, E., Vannucci, G., Anderlini, L. & Bennett, R. A. Kinematics, seismotectonics, and seismic potential of the eastern sector of the European Alps from GPS and seismic deformation data. *Tectonophysics* **688**, 157–181. 10.1016/j.tecto.2016.09.026 (2016).

[79] Sinclair, H. D. Tectonostratigraphic model for underfilled peripheral foreland basins: An Alpine perspective. *Geol. Soc. Am. Bull.* **109**, 324–346. 10.1130/0016-7606(1997)109%3c0324:TMFUPF%3e2.3.CO;2 (1997).

[80] Suppe, J. Kinematics of Arc-Continent Collision, Flipping of Subduction, and Back-Arc Spreading near Taiwan. *Memoir Geol. Soc. China* **6**, 21–33 (1984).

[81] Ustaszewski, K. et al. Crust–mantle boundaries in the Taiwan-Luzon arc-continent collision system determined from local earthquake tomography and 1D models: Implications for the mode of subduction polarity reversal. *Tectonophysics* **578**, 31–49. 10.1016/j.tecto.2011.12.029 (2012).

[82] Replumaz, A. et al. Indian continental subduction and slab break-off during Tertiary collision. *Terra Nova* **22**, 290–296. 10.1111/j.1365-3121.2010.00945.x (2010).

[83] Liang, X. et al. Fragmentation of continental subduction is ending the Himalayan orogeny. *Sci. Bulletin* **68**, 3048–3054. 10.1016/j.scib.2023.10.017 (2023). 10.1016/j.scib.2023.10.017 37919155

[84] Webb, A. A. G. et al. The Himalaya in 3D: Slab dynamics controlled mountain building and monsoon intensification. *Lithosphere* **9**(4), 637–651. 10.1130/L636.1 (2017).

[85] Liang, S. et al. Abrupt topographic descent at the eastern end of the Himalayan orogen: Insights from geodetic analyses. *J. Asian Earth Sci.*10.1016/j.jseas.2024.106300 (2024).

